# Thymoma‐associated autoimmune encephalitis: Analysis of factors determining prognosis

**DOI:** 10.1111/cns.14166

**Published:** 2023-03-13

**Authors:** Wenli Song, Keru Li, Jiao Li, Xiaoni Liu, Xiaoke Wu, Xiaodong Xu, Kangping Xiong, Xiangjun Chen, Yanlin Zhang

**Affiliations:** ^1^ Department of Neurology and Clinical Research Center of Neurological Disease The Second Affiliated Hospital of Soochow University Suzhou China; ^2^ Department of Neurology, Huashan Hospital and Institute of Neurology Fudan University Shanghai China; ^3^ National Center for Neurological Disorders Fudan University Shanghai China; ^4^ Human Phenome Institute Fudan University Shanghai China

**Keywords:** autoimmune encephalitis, immune disorders, prognosis, thymoma, treatment

## Abstract

**Introduction:**

Autoimmune encephalitis (AE) is a heterogeneous group of inflammatory central nervous system disorders caused by a misdirected immune response against self‐antigens expressed in the central nervous system. The thymus is a central organ in the immune system and thymic tumors are thought to be possible initiators of many neurological disorders. Recently, there is growing evidence that thymomas are associated with autoimmune encephalitis.

**Aims:**

Our study initially explored the characteristics of patients with autoimmune encephalitis combined with thymoma.

**Methods:**

We used patient data from January 1, 2011 to October 1, 2021 from the PubMed, Web of Science, Ovid, and CNKI platforms to analyze overall demographics, frequency of symptoms and associations, and treatment prognosis outcomes.

**Results:**

A total of 68 patients were included. There were 39 female cases (57.4%). The mean age was 50 years (IQR 40–66 years). All had acute and subacute onset. The clinical manifestations were mostly cognitive changes (70.6%), mental disorders (57.4%), and epilepsy (50.0%). The most common neuronal antibody was alpha‐amino‐3‐hydroxy‐5‐methyl‐4‐isoxazolepropionic acid (AMPA). Magnetic resonance imaging (MRI) abnormalities were present in 81.0% of patients, mostly in the hippocampus, temporal lobe, and some in cortical and subcortical areas. Abnormalities in the electroencephalogram (EEG) in 69.8% of patients. Treatment involved immunotherapy and thymoma treatment, with 79.7% of patients improving after treatment. While 20.3% of patients had a poor prognosis. Further, 14.8% of patients relapsed. Mental disorders, autonomic dysfunction, sleep disturbances, anti‐Ma2, and thymoma untreated were more frequent in patients with poor prognosis.

**Conclusion:**

Thymoma‐associated autoimmune encephalitis is a unique disease entity. Long‐term follow‐up of chest CT findings is recommended for patients with autoimmune encephalitis.

## Graphical abstract

Patients with AE combined with thymoma tend to present with cognitive changes, epilepsy, and psychobehavioral abnormalities, and the associated antibodies can involve a variety of antibodies, with AMPA being the most common. The overall prognosis of the disease is good. Patients with sleep disturbances, autonomic dysfunction, mental disorders, and anti‐Ma2 do not seem to benefit from treatment. Long‐term follow‐up of chest CT in patients with autoimmune encephalitis is recommended.

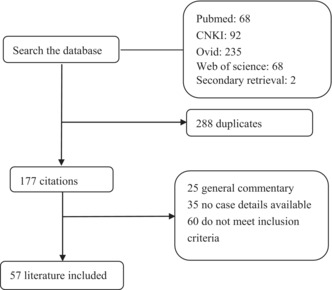

## 1 INTRODUCTION

Autoimmune encephalitis refers to a group of encephalitis mediated by autoimmune mechanisms, it is a potentially treatable condition^1^ and accounts for 20% of encephalitis.^2^ The lesions mainly involve the limbic system. Individuals affected by autoimmune encephalitis usually present with rapidly progressive cognitive impairment, psychiatric symptoms, seizures, dystonia, ataxia, sleep disorders, autonomic dysfunction, etc. The clinical features and prognosis of autoimmune encephalitis vary greatly among individuals, and their immune initiation mechanism is still unknown. Thymoma is a common anterior mediastinal tumor, which is frequently associated with autoimmune disease, mainly myasthenia gravis (MG). About 10%–15% of MG patients have a combination of thymoma.^3^ To date, there are an increasing number of reports suggesting that thymoma is associated with autoimmune encephalitis.

The first case of thymoma‐associated AE was reported by McArdle et al.^4^ in 1988, since then, the disease has attracted special attention, and related cases have been reported. In 2011, a review^5^ of cases of thymoma‐associated paraneoplastic encephalitis (TAPE) reported from 1950 to 2010 indicated that immunotherapy and thymus surgery were the mainstays of good prognosis. Recently, the Japanese Mar Guasp team^6^ retrospectively summarized the clinical outcome and neuroimaging features of antibodies in 43 patients of thymoma‐associated autoimmune encephalitis and analyzed the MRI manifestations associated with each type of antibody and prognosis, but they did not pay attention to the prognostic impact of immunotherapy, tumor interventions and the antibodies associated with a greater risk of disease recurrence. Currently, such studies are rare and results are often conflicting, this forms the basis of this systematic review. This article provides more updated and detailed information on this rare disease by collecting case reports in the last 10 years, analyzing the basic clinical features, laboratory tests, and imaging characteristics, while further analyzing the internal relevant therapeutic and antibody factors affecting prognosis and exploring the magnitude of the clinical value of thymic interventions for the patients, and ultimately to provide clinical guidance.

## 2 MATERIALS AND METHODS

### 2.1 Literature search

The literature search follows the preferred reporting items and source analysis guidelines of the systematic review as far as possible. The search strategy included primary and secondary searches. We performed a literature search in PubMed, Web of Science, Ovid, and CNKI for articles published between January 1, 2011, and October 1, 2021, searched by the search terms “encephalitis” and “autoimmune encephalitis,” “paraneoplastic encephalitis,” “limbic encephalitis,” and “thymoma,” “thymic tumor,” “thymic carcinoma” (Screening Strategy Table S1). We screened the titles and abstracts followed by the full texts of potentially relevant articles. The bibliographies of all included papers were also searched to ensure that no other relevant articles were missed.

### 2.2 Literature inclusion and exclusion criteria

Inclusion criteria: case reports involving patients with thymoma‐associated autoimmune encephalitis. Basic information (age at first presentation, gender), clinical presentation (including cognitive impairment, epilepsy, psychobehavioral disorders, motor disorders, altered consciousness, language disorders, autonomic dysfunction, sleep disorders, fever, headache, dizziness) of the original patient was included. The survey also included descriptions of antibodies, cerebrospinal fluid results, electroencephalogram, magnetic resonance imaging (MRI), thymoma‐related findings, treatment, and disease prognosis. Data were combined to determine overall demographics, frequency of symptoms and associations, and diagnostic findings.

Exclusion criteria: studies mentioning patients with thymoma combined with AE but without case details; reviews; articles unrelated to the topic. Symptoms during the disease include any other symptoms after the initial visit. The time course of all symptoms is rarely reported.

### 2.3 Statistical analysis

Statistical analyses were performed using SPSS IBM 26.0. Continuous variables distributed normally were expressed by mean ± standard deviation. Non‐normal data were presented as a median and interquartile range. The Mann–Whitney *U* and Kruskal–Wallis rank sum tests were compared between groups. And the prognostic factors were analyzed by ordered multi‐class Logistw regression. Values of *p* < 0.05 were considered significant.

## 3 RESULTS

**FIGURE 1:**
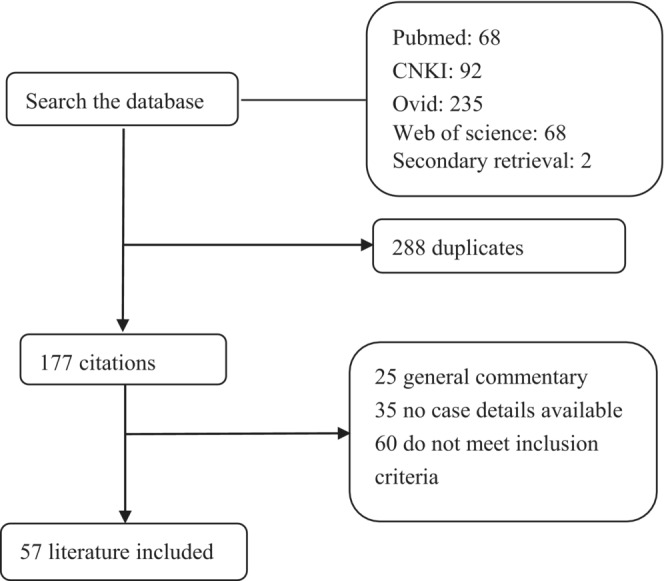
Study flowchart.

The initial search identified 465 articles, of which 407 titles and abstracts were screened and excluded (Figure 1). Fifty‐eight papers^5^, ^7^, ^8^, ^9^, ^10^, ^11^, ^12^, ^13^, ^14^, ^15^, ^16^, ^17^, ^18^, ^19^, ^20^, ^21^, ^22^, ^23^, ^24^, ^25^, ^26^, ^27^, ^28^, ^29^, ^30^, ^31^, ^32^, ^33^, ^34^, ^35^, ^36^, ^37^, ^38^, ^39^, ^40^, ^41^, ^42^, ^43^, ^44^, ^45^, ^46^, ^47^, ^48^, ^49^, ^50^, ^51^, ^52^, ^53^, ^54^, ^55^, ^56^, ^57^, ^58^, ^59^, ^60^, ^61^, ^62^, ^63^ eventually included that enrolled a total of 68 patients.

### 3.1 Clinical features

A total of 68 patients were included in this paper. The median age was 50 years (14–88 years, IQR 40–66 years) and 57.4% were female. History: Some patients had a combination of other systemic immune diseases: myasthenia gravis most, in 19 cases, thyroid disease in four cases, acquired neuromuscular ankylosis, multiple sclerosis, and systemic lupus erythematosus in one case. All patients were combined with thymoma, of which 48 (70.6%) patients' thymoma was found together with autoimmune encephalitis, and in 20 patients (29.4%), thymoma lesions were found first, followed by symptoms of autoimmune encephalitis.

The clinical features are detailed in Table 1 below. Our analysis revealed that the majority of patients presented with cognitive changes (70.6%), mental disorders (57.4%) and epilepsy (50.0%), Other clinical features were also present motor disorders (29.4%), altered consciousness (29.4%), language disorders (25.0%), autonomic dysfunction (19.1%), sleep disorders (13.2%), fever (4.4%), headache (4.44%), dizziness (4.4%). One case reported only the presence of progressive encephalopathy,^18^ one case only encephalitis, with no specific description of the symptom profile.^50^ There were 61 cases with more than two symptoms. The majority of reported cases had an admission modified Rankin scale (mRs) score of 4–5.

**TABLE 1:** Clinical features and ancillary investigations in patients with thymoma‐associated autoimmune encephalitis.

Variable (*n*, %)	Total	Female (*n* = 39)	Man (*n* = 29)	*p*
Age (years)	50 (40–66)	50 (42–66)	50 (36–68)	0.790
Clinical features				
Cognitive impairment	48 (70.6%)	31 (79.5%)	17 (58.6%)	0.105
Mental disorders	39 (57.4%)	23 (59.0%)	16 (55.2%)	0.807
Epilepsy	34 (50.0%)	16 (41.0%)	18 (62.1%)	0.141
Motor disorders	20 (29.4%)	9 (23.1%)	11 (37.9%)	0.282
Altered consciousness	20 (29.4%)	13 (33.3%)	7 (24.1%)	0.436
Language disorders	17 (25.0%)	9 (23.1%)	8 (27.6%)	0.779
Autonomic dysfunction	13 (19.1%)	8 (20.5%)	5 (17.2%)	1.000
Sleep disorders	9 (13.2%)	4 (10.3%)	5 (17.2%)	0.481
Fever	3 (4.4%)	2 (5.1%)	1 (3.4%)	1.000
Headache	3 (4.4%)	2 (5.1%)	1 (3.4%)	1.000
Dizziness	3 (4.4%)	1 (2.6%)	2 (6.9%)	0.571
Combined with other systemic immune diseases	21 (30.9%)	13 (33.3%)	8 (27.6%)	0.791
Myasthenia gravis	18 (26.5%)	11 (28.2%)	7 (24.1%)	0.786
Thyroid disease	4 (5.9%)	3 (7.7%)	1 (3.4%)	0.631
Acquired neuromuscular ankylosis	1 (1.5%)	0 (0.0%)	1 (3.4%)	0.426
Multiple sclerosis	1 (1.5%)	1 (2.6%)	0 (0.0%)	1.000
Systemic lupus erythematosus	1 (1.5%)	1 (2.6%)	0 (0.0%)	1.000
Prior thymoma	20 (29.4%)	13 (33.3%)	7 (24.1%)	0.436
Autoantibodies	56 (86.2%)	32 (86.2%)	25 (85.7%)	1.000
AMPA	19 (28.4%)	9 (23.7%)	10 (34.5%)	0.415
CV2/CRMP5	10 (14.9%)	5 (13.2%)	5 (17.2%)	0.736
LGI1	8 (11.9%)	2 (5.3%)	6 (20.7%)	0.068
GABAa	7 (10.4%)	6 (15.8%)	1 (3.4%)	0.129
VGKC	6 (9.0%)	2 (5.3%)	4 (13.8%)	0.391
CASP	6 (9.0%)	3 (7.9%)	3 (10.3%)	1.000
GABAb	5 (7.5%)	2 (5.3%)	3 (10.3%)	0.645
GAD65	5 (7.5%)	4 (10.5%)	1 (3.4%)	0.379
Titin	4 (6.0%)	2 (5.3%)	2 (6.9%)	1.000
NMDA	2 (3.0%)	1 (2.7%)	1 (3.4%)	1.000
Ma2	2 (3.0%)	2 (5.3%)	0 (0.0%)	0.502
Hu	1 (1.5%)	0 (0.0%)	1 (3.4%)	0.433
AchR	16 (23.9%)	9 (23.7%)	7 (24.1%)	1.000
Ancillary investigations				
CSF	21 (44.7%)	9 (37.5%)	12 (52.2%)	0.385
MRI	51 (81.0%)	30 (85.7%)	21 (75.0%)	0.343
EEG	37 (69.8%)	20 (64.5%)	17 (77.3%)	0.376
Immunotherapy	56 (94.9%)	29 (90.6%)	27 (100.0%)	0.243
Steroids	46 (80.7%)	24 (77.4%)	22 (84.6%)	0.738
IVIg	28 (49.1%)	10 (32.3%)	18 (69.2%)	0.008*
PE	12 (20.7%)	8 (25.0%)	4 (15.4%)	0.518
First‐line treatment	55 (94.8%)	29 (90.6%)	26 (100.0%)	0.245
Second‐line treatment	9 (15.5%)	4 (12.5%)	5 (19.2%)	0.717
Thymoma treatment	54 (90.0%)	30 (90.9%)	24 (88.9%)	1.000
Surgery	54 (90.0%)	30 (90.9%)	24 (88.9%)	1.000
Radiotherapy	9 (15.3%)	7 (21.9%)	2 (7.4%)	0.160
Prognosis	51 (79.7%)	28 (77.8%)	23 (82.1%)	0.761
Recurrence	9 (14.8%)	5 (15.2%)	4 (14.3%)	1.000

* *p* < 0.5.

### 3.2 Ancillary examinations

Lumbar puncture and cerebrospinal fluid examination: lumbar puncture was performed in 67 patients. The results of cerebrospinal fluid examination were not available in 22 patients, abnormal cerebrospinal fluid indices were found in 21 (44.7%) patients. In 26 patients, the cerebrospinal fluid findings were at normal levels. In 16 patients, the white blood cell count was elevated (7–97, in some cases, the data were not available and only indicated elevation) and showed signs of inflammation in the cerebrospinal fluid. In 10 patients, protein was elevated (51–92 mg/dl).

Antibodies: there were 19 positives for amino‐3‐hydroxy‐5‐methyl‐4‐isoxazole propionic acid receptor (AMPAR), 10 positive for anti‐collapsin response mediator protein 5 (CRMP5), eight positives for leucine‐rich, glioma inactivated 1 (LGI1), seven positives for a gamma‐aminobutyric acid receptor A (GABAAR), six positives for contactin‐associated protein‐like 2 (CASPR2) and anti‐voltage‐gated potassium channel (VGKC), five positive for gamma‐aminobutyric acid receptor B (GABABR) and Glutamic Acid Decarboxylase 65 (GAD65), four positive for Myosin (Titin), two positive for N‐methyl‐D‐Aspartate Receptor (NMDAR) and Ma2, one positive for Type 1 antineuronal nuclear antibody (ANNA‐1/“anti‐Hu”), and16 patients with combined Acetylcholine Receptor (AChR). More than one antibody was present in 15 of the 58 patients.

Magnetic resonance imaging (MRI) findings: definitive cranial MRI findings were reported in 66 patients. 51 (81.0%) had abnormalities on MRI, with most of the MRI lesions involving the hippocampus and temporal lobe regions, some involving cortical and subcortical areas.

Electroencephalogram (EEG): An abnormal EEG was shown in 69.8% of patients, mostly showing slow wave and sharp wave emission of frontotemporal lobe.

### 3.3 Treatment and prognosis

Of the 68 patients, the specific treatment plan of 10 patients was unknown, while the remaining 56 patients were treated with first‐line immunotherapy (hormone, gamma globulin, plasmapheresis), treatment modalities are shown in Table 2. After receiving first‐line treatment, most patients observed an improvement in the symptoms of the disease, such as a reduction in the frequency and severity of seizures or a recovery of consciousness. After the first‐line treatment was not improved in some patients, the symptoms improved with the increase in second‐line immunotherapy. Fifty‐four (90.0%) chose thymoma intervention therapy (surgery or radiotherapy and chemotherapy) and pathological findings involving types ab, b1, b2, and b3, which were not specific.

**TABLE 2:** Results from the univariate analysis of clinical data of two groups.

Variable (*n*, %)	Good‐prognosis (*n* = 51)	Poor‐prognosis (*n* = 13)	*p*
Sex (female)	28 (54.9%)	8 (61.5%)	0.761
Age (years)	50 (39–66)	55 (41–70)	0.483
Clinical features			
Cognitive impairment	34 (66.7%)	10 (76.9%)	0.739
Mental disorders	25 (49.0%)	11 (84.6%)	0.028*
Epilepsy	26 (51.0%)	5 (38.5%)	0.539
Motor disorders	18 (35.3%)	2 (15.4%)	0.201
Altered consciousness	14 (27.5%)	4 (30.8%)	1.000
Language disorders	14 (27.5%)	3 (23.1%)	1.000
Autonomic dysfunction	7 (13.7%)	6 (46.2%)	0.018*
Sleep disorders	3 (5.9%)	5 (38.5%)	0.007*
Fever	3 (5.9%)	0 (0.0%)	1.000
Headache	3 (5.9%)	0 (0.0%)	1.000
Dizziness	3 (5.9%)	0 (0.0%)	1.000
Combined with other systemic immune diseases	15 (29.4%)	5 (38.5%)	0.523
Myasthenia gravis	14 (27.5%)	3 (23.1%)	1.000
Thyroid disease	3 (5.9%)	1 (7.7%)	1.000
Acquired neuromuscular ankylosis	1 (2.0%)	0 (0.0%)	1.000
Multiple sclerosis	0 (0.0%)	1 (7.7%)	0.203
Systemic lupus erythematosus	0 (0.0%)	1 (7.7%)	0.203
Prior thymoma	13 (25.5%)	3 (23.1%)	1.000
Autoantibodies	41 (83.7%)	12 (92.3%)	0.670
AMPA	14 (27.5%)	3 (23.1%)	1.000
CV2/CRMP5	7 (13.7%)	3 (23.1%)	0.411
GABAa	7 (13.7%)	0 (0.0%)	0.328
LGI1	7 (13.7%)	1 (7.7%)	1.000
VGKC	5 (9.8%)	2 (15.4%)	0.623
CASP	3 (5.9%)	2 (15.4%)	0.266
GABAb	4 (7.8%)	1 (7.7%)	1.000
GAD65	4 (7.8%)	1 (7.7%)	1.000
Titin	4 (7.8%)	0 (0.0%)	0.574
NMDA	2 (3.9%)	0 (0.0%)	1.000
Hu	1 (2.0%)	0 (0.0%)	1.000
Ma2	0 (0.0%)	2 (15.4%)	0.039*
AchR	13 (25.5%)	2 (15.4%)	0.715
Ancillary investigations			
CSF	13 (38.9%)	6 (60.0%)	0.292
MRI	38 (82.6%)	11 (84.6%)	1.000
EEG	25 (65.8%)	8 (72.7%)	1.000
Immunotherapy	43 (95.6%)	12 (92.3%)	0.540
Steroids	37 (82.2%)	9 (75.0%)	0.683
IVIg	21 (46.7%)	7 (58.3%)	0.530
Steroids + IVIg	17 (37.8%)	5 (38.5%)	1.000
PE	8 (17.8%)	3 (25.0%)	0.683
Second‐line treatment	7 (15.6%)	2 (16.7%)	1.000
Thymoma treatment	46 (95.8%)	8 (66.7%)	0.012*
Surgery	46 (95.8%)	8 (66.7%)	0.012*
Radiotherapy	6 (12.8%)	3 (25.0%)	0.369
Recurrence	8 (16.0%)	1 (9.1%)	1.000

* *p* < 0.5.

The symptoms of 51 (79.7%) patients were improved after treatment (in the literature, some of the improvements are considered to be a decrease in MRS scores, and some cases only describe improvements in clinical manifestations after treatment). Five cases died: one case died of pneumonia, one case refused further treatment of thymoma, one case died of secondary serious complications, and two cases died of progressive thymoma. During follow‐up, nine cases (14.8%) relapsed.

Statistical analysis of patients of different genders showed that the utilization rate of gamma globulin (*p* = 0.008) in male patients was higher than that in female patients. Univariate analysis showed that there were significant differences in clinical manifestations and auxiliary examination values between the good prognosis group and the poor prognosis group. Table 2 summarizes the comparison between patients with good and poor clinical results. Compared with the good prognosis group, the poor prognosis group had a higher incidence of mental disorders (*p* = 0.028), autonomic nervous dysfunction (*p* = 0.018) and sleep disorders (*p* = 0.007), anti‐Ma2 (*p* = 0.039), and less intervention treatment of thymoma (*p* = 0.012).

## 4 DISCUSSION

The thymus, which is located in the anterosuperior mediastinum, is one of the vital organs in the development of the immune system. It is also the site of the production of immune cell T cells. Although it is generally believed that thymus function declines with age, some findings suggest that thymus activity can be maintained in adulthood, which helps explain why thymomas are associated with diseases involving autoimmunity.^64^ Thymomas are thought to be a possible initiator of many neurological disorders.^24^ The specific pathogenesis is currently subjected to the following types of hypotheses: (1) escape theory, thymoma retains some of the functions of thymic cortical epithelial cells. They are capable of inducing T‐cell differentiation and development. However, because thymomas lack a medullary component, T cells that have undergone positive selection cannot undergo effective negative selection. Failure of thymus negative selection causes CNS‐reactive T cells to escape to the periphery, resulting in immune disease.^65^ (2) In genetic theory, genetic changes in thymoma cells affect T cells, resulting in the production of T lymphocytes directed against autoantibodies.^66^ (3) Combined cellular and humoral immunity mechanism theory^67^: auto‐reactive T cells in thymoma leave the thymus and enter the periphery, activating CD4^+^ T cells to complete the conversion from cellular to humoral immunity, thus activating B cells to produce auto‐antibodies. Antigen–antibody binding affects the alteration of signal transduction pathways and the corresponding clinical manifestations. Patients with thymoma can produce a variety of abnormal antibodies that constitute a complex immune system.^48^ Associated paraneoplastic syndromes are involved in several areas, such as myasthenia gravis, optic neuromyelitis optica, autoimmune encephalitis, and subacute cerebellar degeneration and so on.^66^ The autoantibody titer was decreased in some patients after thymoma resection.^68^

In this study, we found that the proportion of men and women with thymoma combined with autoimmune encephalitis was not significantly different and was mostly seen in middle‐aged people. Similar to previous studies, the most common clinical manifestations of thymoma‐associated AE included cognitive dysfunction, mental disorders, epilepsy, and a range of positive antibodies and variable responses to treatment,^43^ suggesting that thymoma‐associated autoimmune encephalitis is a distinct disease entity.

This group of patients may have multiple autoantibodies. In this review, AMPAR was the most common antibody, mediating most of the rapid excitatory neurotransmission in the brain^24^ and is often associated with tumors.^69^ A review published last year^70^ found that patients with thymoma accounted for the largest proportion of patients with tumor who were also positive for AMPAR antibodies, suggesting that patients presenting with these antibodies should be routinely screened for thymus CT. The second antibody in this article is the anti‐VGKC. Previously it was thought that many of the clinical manifestations of autoimmune encephalitis were associated with VGKC antibodies. This class of antibodies is a trans‐synaptic protein adhesion molecule that mainly affects excitatory transmission between neurons.^71^ But later it was found that the three subgroups of VGKC positivity were different in nature^72^ and that the centralized terminology of VGKC‐complex antibodies was replaced. The caspr2 antibody correlates most strongly with thymoma and does not appear to be strongly associated with other tumors such as lung adenocarcinoma.^73^ LGI1‐positive patients rarely have a combination of tumor and the prevalence is at ~10%,^72^ which seems inconsistent with the findings of this paper. We consider the possibility of a selection bias due to this. CV2 protein, also known as CRMP5 protein, is the third most common antibody species, and mainly regulates cell development in the central nervous system. It is also involved in the proliferation of tumor cells. This group of patients is generally considered to be vulnerable to the development of myasthenia gravis and their clinical prognosis is relatively poor.^17^ In this paper, due to sample size limitations, no correlation was shown. GABAA is the fourth most common antibody in this article. Previous studies found that nearly 40% of patients with GABAA had a tumor in combination with thymoma, and the most commonly associated tumor was thymoma.^16^ Antibodies in thymoma combined with autoimmune encephalitis patients also involve anti‐GABAB, GAD65, NMDA, Ma2, Titin, and Hu. Some patients combined more than one antibody, and in these previously reported cases, it is unclear which antibodies were pathogenic and which were concomitant as a review of the associated antibodies did not show a clear pattern.^74^ The thymus is an important immune organ in the human body, its abnormal function can lead to immune dysfunction and the coexistence of multiple autoantibodies in the same patient, which makes the diagnosis and treatment of the disease difficult. It is important to clarify the responsible antibodies.^75^ That needs further exploration of the relationship between various types of antibodies and clinical types in order to clarify the culprit antibody and treat them precisely.

We found that AchR is strongly associated with patients with thymoma‐associated AE. Since MG occurs in 30%–45% of patients with thymoma and the vast majority of MGs with combined thymoma are positive for AchR antibodies,^76^ the high level of AchR antibodies can be explained.

The majority of patients have abnormal EEG, some of whom do not have symptomatic seizures, suggesting that routine screening for EEG indicators is also indicated in patients without seizures. There is no significant difference on MRI between the presentation and that of the autoimmune encephalitis. Most patients have localized imaging, with the lesions mostly involving the temporal lobe of the hippocampus and some accumulating in cortical and subcortical areas.

In the majority of patients, the neurological symptoms of autoimmune encephalitis precede thymoma, and there is a strong correlation between the two, which is currently thought to be due to the microenvironment of the tumor allowing the escape of auto‐reactive T cells, mediating the development of autoimmune disease.^65^ There are still patients with thymoma findings preceding neurological defects, some of whom may develop autoimmune encephalitis after resection. At present, no factors have been identified for the development of disease after thymectomy, whether the thymus can express autoantigens after resection, whether the antigens have colonized the central nervous system before surgery and how they trigger immune disturbances, remains inconclusive and requires further study.

The overall prognosis is good. Treatment involved both immunotherapy and thymoma treatment. The majority of patients improved after first‐line treatment, with some experiencing poor outcomes and recurrence. Drug use is generally associated with doctor habits, but we found that the use of gamma globulin is higher in male patients, and the specific cause of this phenomenon remains unknown, which may be related to epilepsy. The symptoms of most patients were improved after thymus intervention, consistent with the previous studies,^64^ suggesting that thymectomy is beneficial for recovery and prognosis. Routine screening for tumor indicators is necessary. The presence of thymoma reignition in some cases and the remission of new neurological symptoms after reoperation suggest that patients with thymoma still need a regular review of thymic tissue for time management.

In this article, we found that the treatment of the patients who exhibit sleep disturbances does not seem to provide a benefit, as are autonomic dysfunction and mental disorders, which require further attention. Sleep disorders are generally common in neurodegenerative diseases such as Alzheimer's disease and Parkinson's disease. At present, the relationship between autoimmune encephalitis and sleep disorders has been gradually concerned. At present, the pathophysiological significance between autoimmune encephalitis and sleep disorders is complex, and there is no exact mechanism.^77^ A study^78^ shows that sleep disorders are very common in AE. Improving the identification and treatment of sleep disorders can improve the long‐term prognosis. Although this article shows that there is a correlation between sleep disorder and prognosis, the collection of data is limited by the author of the case report, the accuracy is not high, and further prospective observation and study are needed. Autonomic nervous dysfunction is also one of the common clinical features of autoimmune encephalitis, which is characterized by the dysfunction of cardiovascular, digestive, and endocrine systems. This paper shows that this feature is related to the poor prognosis of patients with autoimmune brain complicated with thymoma. One case^79^ has been reported that it can threaten the survival of patients. However, previous studies^80^ have shown that this symptom is a favorable factor for the prognosis of autoimmune brain patients, which needs to be further discussed. Autoimmune encephalitis is mainly characterized by mental or neurocognitive syndrome, benefiting 94% of patients treated with immunomodulators.^81^ Ma2 is a class of rare onconeural antibodies mostly associated with testicular, lung, and gastrointestinal tumors.^82^ Previous studies have shown that Ma2‐paraneoplastic neurologic syndromes (PNS) has a high mortality rate and poor prognosis even after tumor treatment and immunosuppressive therapy.^8^ This article inductively collects analytically agree with the view. However, due to too few cases, inductive bias may exist, which needs to be verified by further prospective studies. This paper found that among the patients with thymoma, the prognosis of the patients with this manifestation was poor, suggesting that attention should be paid to these patients and timely intervention and treatment. The poor prognosis of the patients without timely thymus intervention was relatively large, and the side showed the superiority of thymus therapy.

Our review highlights the need for future research, particularly to assess the impact of specific clinical presentations, comorbid immune diseases, and surgical interventions on prognosis and recurrence. Future attention will be focused on this important modifiable factor that alters patient prognosis through further prospective cohort studies. In addition, randomized controlled trials comparing immunotherapy and thymic interventions are needed in this field to develop treatment guidelines for this patient group.

## 5 RESEARCH LIMITATIONS

The present study has some limitations. First, retrospective reviews can lead to bias. Clinical case reports differ in the reporting of patient information, treatment, and prognosis. Second, the study only examined reports published from January 1, 2011 to October 1, 2021. In addition, although the search for autoimmune encephalitis and thymoma through the database was the basis of this study, there may still be some potential patient information that was missed, limiting the breadth of data collected. Third, the search was limited to published articles, which may have caused publication bias or overestimated the associations. This is variability with clinical reality.

## 6 CONCLUSION

In summary, patients with AE combined with thymoma tend to present with cognitive changes, epilepsy, and mental disorders, and the associated antibodies can involve a variety of antibodies, with AMPA being the most common. The overall prognosis of the disease is good. Patients with sleep disturbances, autonomic dysfunction, mental disorders, and anti‐Ma2, without timely thymus intervention do not seem to benefit from treatment. Long‐term follow‐up of chest CT in patients with autoimmune encephalitis is recommended.

## CONFLICT OF INTEREST STATEMENT

The authors declare no conflict of interest.

## Supporting information


Table S1

## Data Availability

The data that support the findings of this study are available from the corresponding author upon reasonable request.
