# Supplementary material for: Thymoma‐associated autoimmune encephalitis: Analysis of factors determining prognosis
Source: CNS Neurosci Ther. 2023 Mar 13;29(5):1213–21. doi: 10.1111/cns.14166 (PMC10068466; doi:10.1111/cns.14166)
Supplement: Supplementary file 1 — Table S1 [file CNS-29-1213-s001.docx]

**Table S1 Search strategy**

| **English literature search dates** | | |
| --- | --- | --- |
| Encephalitis | Autoimmune encephalitis  Paraneoplastic encephalitis  Limbic encephalitis  Anti NMDA  Anti VGKC  Anti LGI1  Anti CASPR2  Anti AMPA  Anti GAD65  Anti GABA  Anti GABAA  Anti GABAB  Anti D2  Anti Dopamine-2  Anti DPPX  Anti Glycine  Anti GlyR  Anti Glutamate  Anti mGluR1  Anti mGluR5  Anti amphiphysin  Hashimoto  Anti Neuronal  Anti Hu  Anti Yo  Anti Ri  Anti CV2  Anti CRMP5  Anti Ma  Anti Recoverin  Anti Tr  Anti Ta  ANNA-1  ANNA-2  ANNA-3  Anti Purkinje | **Thymoma**  **Thymomas**  **Carcinoma, Thymic**  **Carcinomas, Thymic**  **Thymic Carcinoma**  **Thymic Carcinomas** |

| **Database** | **Search strategy** | **Result** |
| --- | --- | --- |
| PUBMED | **((Encephalitis[Title/Abstract]) AND ((((((((((((((((((((((((((((((((((((Autoimmune encephalitis[Title/Abstract]) OR (Paraneoplastic encephalitis[Title/Abstract])) OR (Limbic encephalitis[Title/Abstract])) OR (Anti NMDA[Title/Abstract])) OR (Anti VGKC[Title/Abstract])) OR (Anti LGI1[Title/Abstract])) OR (Anti CASPR2[Title/Abstract])) OR (Anti AMPA[Title/Abstract])) OR (Anti GAD65[Title/Abstract])) OR (Anti GABA[Title/Abstract])) OR (Anti GABAA[Title/Abstract])) OR (Anti GABAB[Title/Abstract])) OR (Anti D2[Title/Abstract])) OR (Anti Dopamine-2[Title/Abstract])) OR (Anti DPPX[Title/Abstract])) OR (Anti Glycine[Title/Abstract])) OR (Anti GlyR[Title/Abstract])) OR (Anti Glutamate[Title/Abstract])) OR (Anti mGluR1[Title/Abstract])) OR (Anti mGluR5[Title/Abstract])) OR (Anti amphiphysin[Title/Abstract])) OR (Hashimoto[Title/Abstract])) OR (Anti Neuronal[Title/Abstract])) OR (Anti Hu[Title/Abstract])) OR (Anti Yo[Title/Abstract])) OR (Anti Ri[Title/Abstract])) OR (Anti CV2[Title/Abstract])) OR (Anti CRMP5[Title/Abstract])) OR (Anti Ma[Title/Abstract])) OR (Anti Recoverin[Title/Abstract])) OR (Anti Tr[Title/Abstract])) OR (Anti Ta[Title/Abstract])) OR (ANNA-1[Title/Abstract])) OR (ANNA-2[Title/Abstract])) OR (ANNA-3[Title/Abstract])) OR (Anti Purkinje[Title/Abstract]))) AND (((((((Thymoma[Title/Abstract]) ) OR (Thymomas[Title/Abstract])) OR (Carcinoma, Thymic[Title/Abstract])) OR (Carcinomas, Thymic[Title/Abstract])) OR (Thymic Carcinoma[Title/Abstract])) OR (Thymic Carcinomas[Title/Abstract])) AND (2011:2022[pdat])** Filters: **from 2011/1/1 - 2021/10/1** | N=68 |
| CNKI | (((old_version_topic='Autoimmune encephalitis' or keyword=xls('Autoimmune encephalitis') or title=xls('Autoimmune encephalitis') or abstract=xls('Autoimmune encephalitis') ) OR (((old_version_topic='Associated carcinomatous encephalitis' or keyword=xls('Associated carcinomatous encephalitis') or title=xls('Associated carcinomatous encephalitis') )) (( old-topic='Thymoma' or keyword=xls('Thymoma') or title=xls('Thymoma') or abstract=xls('Thymoma') ) OR (( old-topic='Thymic tumour' or keyword=xls('Thymic tumour') or title=xls('Thymic tumour') or abstract=xls('Thymic tumours') ) OR (( (old version subject='Thymic cancer' or keyword=xls('Thymic cancer') or title=xls('Thymic cancer') or abstract=xls('Thymic cancer') )) AND ((date of publication between (' 2011.1.1', '2021.10.1')) | N=92 |
| Ovid | （Database Field Guide Books@Ovid Full Text, Database Field Guide Journals@Ovid, Database Field Guide Fudan University - Full Text Journals, Database Field Guide JBI EBP Database Current to November 02, 2022, Database Field Guide BIOSIS Previews 1994 to 2022 Week 50, Database Field Guide Embase 1974 to 2022 November 07, Database Field Guide Global Health Archive 1910 to 1972, Database Field Guide Ovid MEDLINE(R) ALL 1946 to November 07, 2022）  #1 Encephalitis.ab. (N=117898)  #2(Thymoma or Thymomas or Carcinoma, Thymic or Carcinomas, Thymic or Thymic Carcinoma or Thymic Carcinomas).ab.(N=26809)  #3 (Autoimmune encephalitis or Paraneoplastic encephalitis or Limbic encephalitis or Anti NMDA or Anti VGKC or Anti LGI1 or Anti CASPR2 or Anti AMPA or Anti GAD65 or Anti GABA or Anti GABA or Anti GABAB or Anti D2 or Anti Dopamine-2 or Anti DPPX or Anti Glycine or Anti GlyR or Anti Glutamate or Anti mGluR1 or Anti mGluR5 or Anti amphiphysin or Hashimoto or Anti Neuronal or Anti Hu or Anti Yo or Anti Ri or Anti CV2 or Anti CRMP5 or Anti Ma or Anti Recoverin or Anti Tr or Anti Ta or ANNA-1 or ANNA-2 or ANNA-3 or Anti Purkinje).ab.(n= 25752)  #4 #1 and #2 and #3 (n=410)  #5 2011.1.1-2021.10.1 | N=235 |
| Web of science | #1 Encephalitis.ab. (N=43125)  #2(Thymoma or Thymomas or Carcinoma, Thymic or Carcinomas, Thymic or Thymic Carcinoma or Thymic Carcinomas).ab.(N=9964)  #3 (Autoimmune encephalitis or Paraneoplastic encephalitis or Limbic encephalitis or Anti NMDA or Anti VGKC or Anti LGI1 or Anti CASPR2 or Anti AMPA or Anti GAD65 or Anti GABA or Anti GABAA or Anti GABAB or Anti D2 or Anti Dopamine-2 or Anti DPPX or Anti Glycine or Anti GlyR or Anti Glutamate or Anti mGluR1 or Anti mGluR5 or Anti amphiphysin or Hashimoto or Anti Neuronal or Anti Hu or Anti Yo or Anti Ri or Anti CV2 or Anti CRMP5 or Anti Ma or Anti Recoverin or Anti Tr or Anti Ta or ANNA-1 or ANNA-2 or ANNA-3 or Anti Purkinje).ab.(n= 9964)  #4 #1 and #2 and #3 ‘2011.1.1-2021.10.1’(n=68) | N=68 |
